# Genetic tool development and systemic regulation in biosynthetic technology

**DOI:** 10.1186/s13068-018-1153-5

**Published:** 2018-06-01

**Authors:** Zhongxue Dai, Shangjie Zhang, Qiao Yang, Wenming Zhang, Xiujuan Qian, Weiliang Dong, Min Jiang, Fengxue Xin

**Affiliations:** 1 State Key Laboratory of Materials-Oriented Chemical Engineering, College of Biotechnology and Pharmaceutical Engineering, Nanjing Tech University, Puzhu South Road 30#, Nanjing, 211800 People’s Republic of China; 2 Jiangsu National Synergetic Innovation Center for Advanced Materials (SICAM), Nanjing Tech University, Nanjing, 211800 People’s Republic of China

**Keywords:** Synthetic biology, Genetic engineering, Synthetic genomics, Genetic tools

## Abstract

With the increased development in research, innovation, and policy interest in recent years, biosynthetic technology has developed rapidly, which combines engineering, electronics, computer science, mathematics, and other disciplines based on classical genetic engineering and metabolic engineering. It gives a wider perspective and a deeper level to perceive the nature of life via cell mechanism, regulatory networks, or biological evolution. Currently, synthetic biology has made great breakthrough in energy, chemical industry, and medicine industries, particularly in the programmable genetic control at multiple levels of regulation to perform designed goals. In this review, the most advanced and comprehensive developments achieved in biosynthetic technology were represented, including genetic engineering as well as synthetic genomics. In addition, the superiority together with the limitations of the current genome-editing tools were summarized.

## Background

Synthetic biology is based on the study of system biology, using “problem-oriented” and “bottom–up” engineering design ideas to build standardized components and modules, which rebuilds existing natural systems to obtain new biological systems or de novo synthesize new artificial biological systems. Synthetic biology covers a broad research area, which combines the disciplines in biology and engineering. It aims to design and create new biological systems to perform functions that are not found in nature. The statement of “synthetic biology” was first appeared in 1980. Until recently, with the matureness of genetic tools, the rapid growth of synthetic biology in research, innovation, and policy interest has been seen [1]. The opinion was held that synthetic biology will bring to the industries of chemical synthesis (including materials, energy, and natural compounds), medicine, agriculture, and environment a surprising “gift”.

Synthetic biology is deemed as an emerging research field, which will bring new opportunities as well as challenges to biotechnology. Synthetic biology can be classified into three levels. First, new types of regulatory network are constructed using natural biological motifs or modules with known functions. The second is to synthesize genomic DNA and reconstruct the living body using de novo synthesis method. The third is the creation of a completely new biological system and even artificial life after the first two fields have been well developed. By several decades’ development of synthetic biology, great progresses in fundamental science and practical applications have been achieved. Furthermore, new breakthroughs will be brought to the biotechnology in the foreseeable future.

Synthetic biology covers various aspects, including genetic circuits, minimal genomes, DNA synthesis, and genetic modification [2]. And also, synthetic biology can be developed in organisms with reverse genetic tools and methodologies, including genetic transformation, selection markers, specific plasmid vectors, different promoters, and other advanced molecular biology techniques, such as (zinc finger proteins) ZFPs, (transcription activators like effector nucleases) TALENs, and (clustered regularly interspaced short palindromic repeats) CRISPR [3, 4]. To provide insight to further understand synthetic biology and discuss future perspectives in this area, the most updated techniques occurring in synthetic biology are summarized in this review.

## Genetic circuits

In the previous studies, researchers have used genetic elements to develop a variety of genetic circuits, which can be widely applied to cellular regulation process. However, it was constrained in the simple assembly of gene-regulatory parts or modules [5]. Thus, recent research trends in this field have focused on the development of a predictable and quantitative mode [6, 7]. With the deepening of research, more and more cellular machines were developed for controlling gene expression, such as some regulatory motifs including genetic switches, oscillators, amplifiers, promoters, and repressors [8].

### Cellular regulatory mechanisms of genetic circuits

**Fig. 1:**
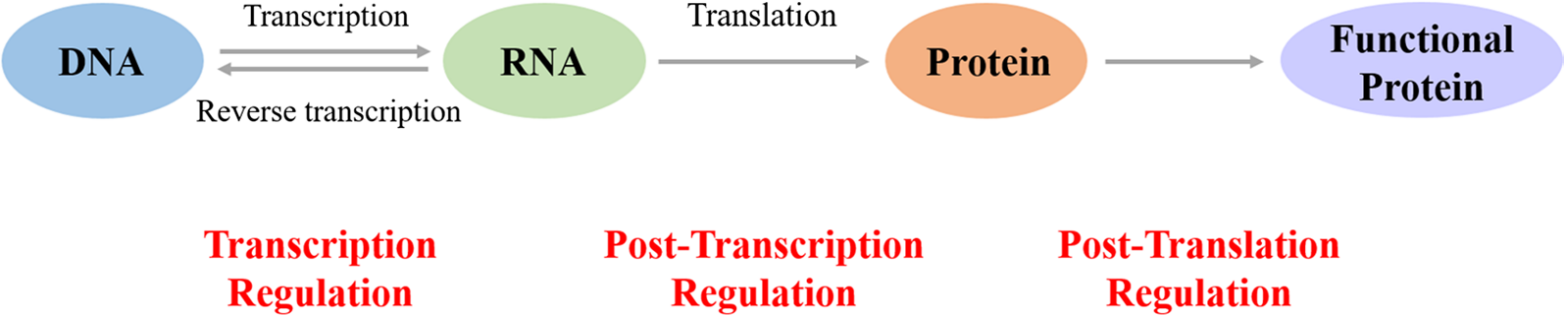
Relation between transcription regulation, post-transcription regulation, and post-translation regulation

Cellular regulation covers a broad range, including transcriptional level, post-transcriptional level, and post-translational level (Fig. 1) [9–11]. Approaches for gene expression at the transcriptional level mainly include synthetic promoter libraries [12, 13], modular system, transcription machinery engineering [14, 15], and transcription factor [16]. These approaches have been wildly used in theory and applications to design and optimize biological systems. Keasling et al. held the opinion that some structural elements for post-transcriptional control can influence protein expression based on a particular mRNA sequence [17]. For example, riboswitches are genetic switches regulating at post-transcriptional levels, which usually exist in the untranslated region of metabolic gene mRNA. Riboswitches possess the abilities of sensing small-molecule metabolites and binding to them, thus alter the secondary structure of RNA to regulate the expression of the corresponding metabolic genes. Hence, riboswitches can be used to design new molecular biosensors [18]. For example, the expression of reporter genes can be regulated by riboswitches to convert enzymatic signals to more detectable ones [19]. Furthermore, riboswitches can also be integrated into more complex gene circuits to achieve regulatory effects [20].

**Fig. 2:**
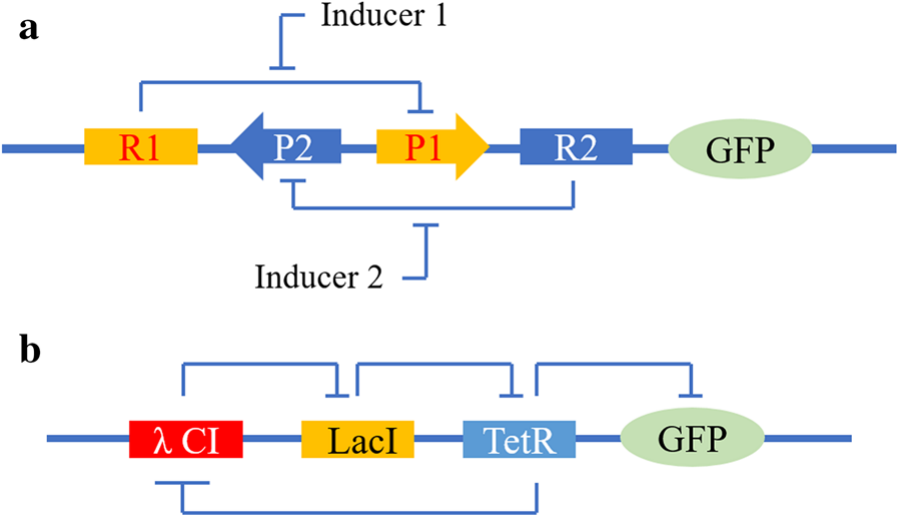
**a** GFP: green fluorescent protein. Toggle switches possess two repressors (R1 and R2) and two promoters (P1 and P2). R1 is transcribed by P2 and can inhibit P1. R2 is transcribed by P1 and can inhibit P2. In addition, R1 is inducted by Inducer1 and R2 is inducted by Inducer2. The transcriptional states can be flipped by adding inducers. **b** LacI inhibits the transcription of TetR, and then TetR inhibits the expression of CI. Finally, CI inhibits LacI expression, LacI inhibits the transcription of TetR, and TetR inhibits the expression of CI and GFP

In addition to genetic switches, more complex genetic switch systems were also developed to program and control the desired electrical output. For instance, a genetic toggle switch was built in *Escherichia coli* inspired by the idea of electronic engineering. As a synthetic and bi-stable gene-regulatory network, the genetic toggle switch is composed of two repressible promoters arranged in a mutually inhibitory network (P1 and P2) and two repressors (R1 and R2) (Fig. 2a). Each promoter is inhibited by the repressor, which is transcribed by the opposing promoter. It presents a near-perfect switching threshold under the fast conversion between stable states using transient chemical or thermal induction [21]. In general, as a practical device, the toggle switch forms a synthetic and addressable cellular memory unit, and has great influence in biotechnology, biocomputing, and gene therapy.

Downstream gene expression can be controlled by placing the proteins’ binding domains within promoter regions. Elowitz and Leibler constructed an oscillating network in *E. coli* with three transcriptional repressor proteins: LacI (the earliest model of gene regulation under the control of lac operon) from *E. coli*, TetR (another model of gene repression) from the tetracycline-resistance transposon Tn10 and CI (a common transcription factor function as a toggle switch) from λ phage (Fig. 2b). LacI inhibits the transcription of TetR, and then TetR inhibits the expression of λCI. Finally, λCI inhibits the function of LacI, which constructs a harmony system with mutual restraint. For a visual readout of the status in individual cells, the green fluorescent protein (GFP) is also added in the system and induced periodically. Due to the tardiness of the generated oscillations than the cell-division cycle with typical periods of hours, the status of the oscillator has to be transmitted from generation to generation [22]. Such “rational network design” may not only lead to the engineering of new cellular behaviors, but also improve the understanding of naturally occurring networks.

### The application of genetic circuits

With the rapid development of synthetic biology over the last several decades, fine-tuning of gene expression has been applied to many organisms and heterologous systems in metabolic engineering and other synthetic biology systems [23–26]. In general, to improve the tailored metabolite production of industrial interest like biofuels or organic acids, etc., the designed or redesigned metabolic pathways have become emphasis in microbes [27]. Researchers have modulated various biomanufacturing-related metabolic pathways originating from different sources, and assembled them in the model organism to obtain suitable biosynthetic pathways. The reconstructed microbes possess increased efficiency of metabolic pathways, which will increase the final product titer, yield, and productivity (TYP), and thus reduce the cost on large-scale production.

For instance, RNA switches have been successfully applied to regulate gene expression and modulate metabolic flux in yeasts [28, 29]. For the purpose of decreasing by-product synthesis, fine-tuning of GPP1 (glycerol-l-phosphatephos-phohydrolase1) and PDC (pyruvate decarboxylase) expression levels were implemented, which are responsible for the production of glycerol and ethanol. Chen et al. constructed two RNA switches to bind different target mRNA: sRNA-RHR2 (tetracycline-responsive GPP1 regulator) and sRNA-PDC6 (theophylline-responsive PDC regulator). The final strain possessed decreased enzyme activities (28.3 and 48.4%) and by-product production (91.9 and 59.5%), respectively. Furthermore, the RNA switches increased fumaric acid production from 28.6 to 33.1 g/L using *Saccharomyces cerevisiae* [30]. These results demonstrated that the insertion of synthetic RNA switches was able to repress the by-product formation without burdening the host cell system. Moreover, RNA switches can be modified to recognize new small molecules with different specificities and mechanisms using other selection strategies. In other work, ligand-responsive RNA switches based on post-transcriptional control were developed in *S. cerevisiae* for the purpose of constructing high-throughput enzyme evolution platform [31].

It is well known that permanent knock-out of undesired genes has a positive effect on improving the titer and yield of the target product. However, deletion of genes related to the cell growth could affect the growth rate, perhaps results in cell death. An alternate approach is to turn these genes off after cells growth reached certain levels, and then inhibit gene expression [32]. As the precursor of isopropanol, acetyl-CoA can be converted to citric acid catalyzed by citrate synthase encoded by *gltA* gene. However, if deletion of *gltA* gene occurred in *E. coli*, the bacterial growth will stop. Thus, a metabolic toggle switch (MTS) was developed by Soma et al. for the purpose of inhibiting *gltA* expression together with keeping good strains growth. After introduction of the *gltA* OFF switch, the expression of *gltA* was turned off and the carbon flux was redirected to isopropanol synthesis, resulting in more than threefold improvement [33]. Several years later, Soma et al. optimized the MTS approach and overexpressed pyruvate oxidase encoded by *poxB* and acetyl-CoA synthase encoded by *acs*, which are responsible for the acetyl-COA synthesis. Promoter P_L_lacO_1_ controls the expression of *poxB* and *acs* genes, while promoter P_L_tetO_1_ controls the repression of TetR. Then, metabolic influx into the TCA cycle could be interrupted. At the same time, isopropanol synthesis was enhanced [34]. These developments illustrate that genetic circuits have tremendous potential for constructing various biological systems with a broad range of practical applications.

Riboswitches are considered as useful tools for monitoring various metabolites due to the ability of sensing specific molecule metabolites and binding to them. As an in vivo metabolite sensor, riboswitch is called RNA biosensor, which can regulates gene expression by changing their conformation upon binding of specific molecules. To enhance the productivity and yield of naringenin, a riboswitch was applied to detect and monitor intracellular or extracellular naringenin. Jang et al. constructed a riboswitch plasmid library and then introduced two in vivo selection routes, which were able to adjust the operational ranges of the riboswitch. Finally, the selected naringenin riboswitch can respond to their ligands faster and eliminate off-target effects [35]. Moreover, an artificial l-tryptophan riboswitch was used to activate gene expression. When adding 1 g/L l-tryptophan, the gene was up-regulated by 1.58-fold compared with no l-tryptophan was added [36].

The other application of genetic circuits is bioremediation. The current environment and ecosystem are greatly suffering from the modernization and industrialization. To deal with this issue properly, the environmental monitoring and remediation systems should be developed urgently [37]. Based on synthetic biology technologies, some advanced biosensors are expected to break down the target molecules [38–40]. Genetic switches can assist programing cells in sensing the multitudinous signals and putting forward some advantageous responses during the complex and uncertain environment [41]. In particular, biosensors fused with synthetic biology technologies show an outstanding performance among the ongoing approaches developed for bioremediation owning to the complement of both laboratory-based and field analytical methods for environmental monitoring. For instance, mercury is widely circulated in industrial processes including material processing, mining, and coal combustion, which damage the water source and food chain seriously [42, 43]. Given this problem, an engineered strain was constructed to sense and sequester Hg^2+^ ions by integrating a mercury-responsive transcriptional regulator (MerR regulator). In addition, this mercury sensor circuit contains cell-surface displayed heavy metal-binding metallothioneins and Hg^2+^ transportation system with the goal of remediating polluted water. When perceiving the presence of mercury, the MerR repressor will change the conformation and bind to Hg^2+^, followed by mercury sequestration [44]. Along with the technical progress, more advanced engineered biosensors may enable the monitor sensors to act as bioreactors to break down target molecules [38]. In general, genetic circuits could be designed to enable the host organisms to act as biosensors and bioreactors, thus to sense and break down environmental pollutants. Undoubtedly, synthetic biology will be a powerful tool to dramatically reduce the environmental pollution in the future.

## Synthetic genomics

From the first International Conference of Synthetic Biology (SB1.0) organized by Massachusetts Institute of Technology(MIT) in 2004 to SB7.0 held in Singapore in 2017, synthetic biology has gradually entered the stage of rapid development [1]. During the early twenty-first century, whole genomes of many species have been sequenced, providing data support for DNA synthesis, which endows the living beings with new genetic traits. Synthetic genomics include plenty of techniques, such as chemical synthesis, genome design, assembly, and transplantation with emphasis on the design and synthesis of the whole genome. As progress moves far ahead of synthetic biology, more and more attentions are paid to the scientific research in synthetic genomics.

### The development of DNA synthesis

**Fig. 3:**
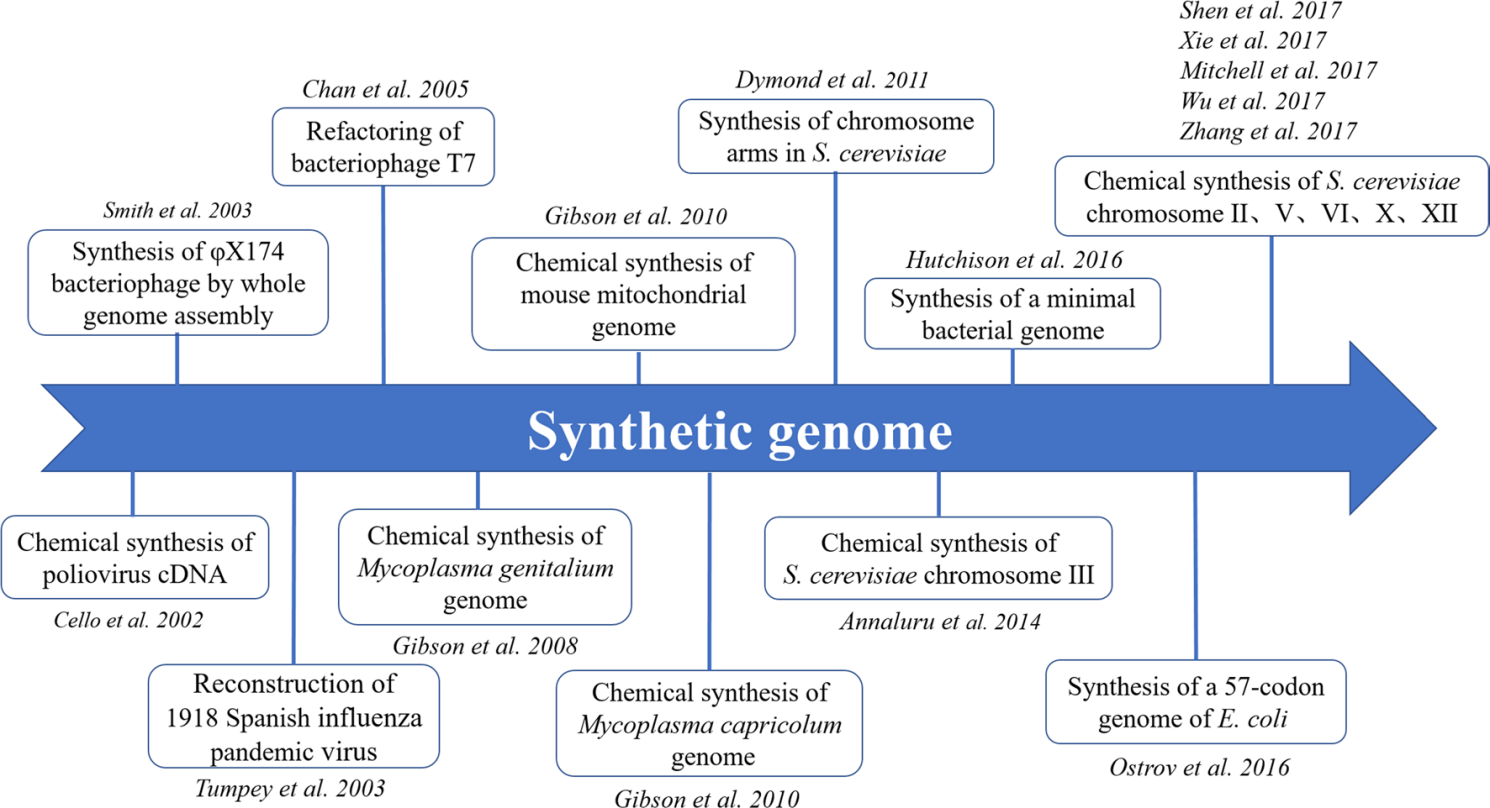
Development of the synthetic genomics

Initially, DNA synthesis belongs to “replication synthesis”, because the biologically active genome is chemically synthesized without major changes to its native genomic sequence (Fig. 3) [45–58]. The main task in this period was to reconstruct the viral genome using reverse genetics. In 2002, Cello et al. assembled the full-length poliovirus complementary DNA (cDNA) using chemically synthesized oligonucleotides of plus and minus strand polarity with the length of 7.5 kb [45]. One year later, Smith et al. chemically synthesized the complete infectious genome of bacteriophage with the length of 5386 bp from a single pool of assembled oligonucleotides [46]. Recently, researchers began to challenge the synthesis of larger scale genomes from a few kb to a few hundred kb owning to the fast-development of synthetic genomics [47, 48]. In 2008, Gibson et al. synthesized the genome of the well-known minimal prokaryotic cell, *M. genitalium*, whose genomes is 582 kb [49]. Two years later, the design, synthesis, and assembly of *Mycoplasma mycoides* JCVI-syn1.0 genome with 1.08-mega-base pairs (1.08 Mbp) were completed [51]. Since then, synthetic genomics entered the “design and synthesis” era, and more genome structures were designed and reconstructed [50]. The representative work includes minimization of *Mycoplasma* genome, recoding *E. coli* genome and artificial synthesis of *S. cerevisiae* chromosome. Especially, in 2016, Venter et al. constructed a smaller genome compared with that of any autonomously replicating cell found in nature by minimizing the synthetic genome of *M. mycoides* JCVI-syn1.0 (1079 kbp) to the JCVI-syn3.0 (531 kbp, 473 genes) [54]. Meanwhile, Ostrov et al. re-encoded the genome of *E. coli* and successfully designed a new genome, which contains only 57 codons [59]. This work underscored the feasibility of rewriting genomes and established a framework for large-scale design, assembly, troubleshooting, and phenotypic analysis of synthetic organisms. In 2009, Dymond et al. proposed the Synthetic Yeast Genome Project (Sc2.0 Project) [52]. Up until now, six chromosomes of *S. cerevisiae* have been successfully designed and synthesized [53, 55, 57, 58, 60, 61].

Currently, the prevailing synthetic methods for DNA synthesis are PCR-based and ligase-based DNA. For better development of synthetic genomics, new synthesis and assembly techniques are needed to meet the increasing demands, and many research groups are devoted to this topic. For example, a PCR-based and two-step DNA synthesis (PTDS) method was modified to synthesize long segments of DNA, which involved the synthesis of individual fragments of interested DNA [62]. 60 mer oligonucleotides with 20 bp overlap can produce 500 bp DNA fragment, and PCR amplification can assemble the entire sequence of interested DNA with two outermost primers. This modified method can produce DNA fragments of 5–6 kb with high G+C contents within 5–7 days [63]. Then, a method of PCR-based accurate synthesis (PAS) of long DNA sequences was assembled. In addition, purification using PAGE and error correction using overlap-extension PCR were added based on the PTDS method, resulting in longer DNA fragments of 12 kb within 7 days [64].

Directed evolution in vitro is another powerful molecular tool for designing new biological parts [65]. DNA synthesis methods are usually used to investigate the function of interested enzymes. Semi-rational design and high-throughput screening together with DNA shuffling and screening were applied for directed in vitro evolution to investigate the function of reporter enzymes [66, 67]. The rapid growth of synthetic biology in DNA synthesis includes the improvements of longer fragments, higher accuracy, and new capabilities, which not only reads, but also edits and rewrites genes and cells of organisms.

### Minimal genomes

Another significant part of synthetic biology is minimal genomes, in which only a minimal DNA sequence is contained for the life maintenance. The ideal minimal genome will only be composed of genes that are essential for the survival of organisms under defined conditions. The non-essential genes and non-encoding regions are usually eliminated, such as genetic elements of alternative metabolic pathways or those encoding responses to stress situations [54]. If microbial chassis only contains the minimum of essential genes, i.e., minimal genome, the production of desired compounds would be more economically feasible. It is believed that minimal cells built on minimal genomes can serve as efficient platforms with new functions. In contrast to traditional recombinant technology, the technique of minimal genomes is far more advanced and directed, rather than the idea of fiddling with a handful of genes to tune one or two metabolic pathways. Most of them adopted top–down approaches for mapping essential and non-essential genes.

In general, natural product discovery was a ‘luck by chance’ event, which was driven by bioactivity-guided chemical screening. Nowadays, novel secondary metabolites can be discovered faster with greater functional abilities via genome mining. Many whole genome-sequencing projects have revealed a number of gene clusters specialized in production of novel chemicals [68]. For example, proteo-bacteria of *Burkholderia*, *Photorhabdus,* and *Xenorhabdus* species are being explored for full extent of secondary metabolite biosynthetic capabilities [69, 70]. Unique gene clusters can be uploaded as heterologous pathways in suitable expression host or chassis for the production of value-added compounds. To this end, it is important to understand the genome structure of the chassis in terms of modularity and essentiality. Thus, the minimal essential gene sets can be identified or filtered.

## Genome-editing tools

Genome engineering has been developed to create industrial strains for various value-added products production. The wide availability of genomic data and gene-editing technologies makes designing orthogonal and heterologous metabolic pathways available [71]. For the time being, several programmable nucleases have been used for genome editing, such as ZFPs, TALENS, and CRISPR/dCas9 systems [72, 73] (Table 1).

**Table 1:** Difference between the modern gene-editing tools ZFNs, TALENs, and CRISPR–Cas9

No.	Gene-editing	Features	Advantages	Limitations	References
1.	ZFNs	Restriction nuclease Fok1 fused to multiple zinc finger peptides, each target triplet codon of genomic DNA Target site length 18–36 base pair Binding specificity–3 nucleotide Nuclease design success rate low Effect of CpG methylation not known	Adequate flexibility Easy gene delivery to the desired target Targeting efficiency variable Recognizing specific long target sequences	Can have high off-target frequency No high-throughput targeting High cost Low specificity and can be influenced by neighboring protein domain easily	[76–78] [81] [83]
2.	TALENs	Non-specific DNA nuclease fused to a domain specific for genomic loci Target site length 30–40 base pair Binding specific-1 nucleotide Nuclease design success rate high Sensitive to CpG methylation	High specific and easy to design High targeting efficiency	Heavier to deliver to the targets Repetitive sequence may cause unintended cuts to the DNA sequence Low off-target effect Limited/low high-throughput targeting	[79, 80] [82] [84–86]
3.	CRISPR–Cas9	20 nucleotide crRNA fused to Cas9 nuclease and tracrRNA Target site length 20–22 base pair Binding specific—1:1 nucleotide Nuclease design success rate high No effect of CpG methylation	High specific and easy multiplexed gene editing High targeting efficiency	Some/variable off-target effect No limitation in high-throughput targeting	[87, 88]

### Summary of genome-engineering tools

**Fig. 4:**
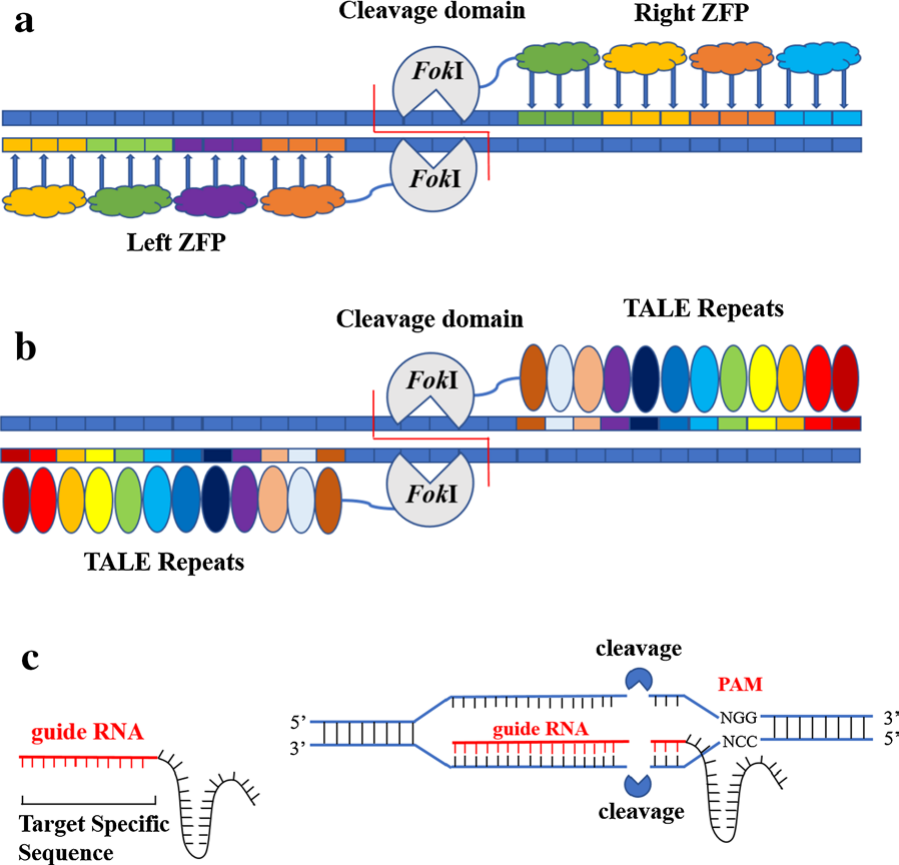
**a** Each ZFN contains the cleavage domain of FokI linked to several zinc fingers which can be designed to specifically recognize that flank the cleavage site. **b** TALEN target sites consist of two TALE binding sites separated by a spacer sequence of varying length. **c** CRISPR–Cas9 is a two-component system composed of Cas9 and gRNA. Once Cas9 finds a PAM site if the gRNA binds to the DNA, a double break occurs three base pairs upstream the PAM

ZFNs and TALENs are artificially designed restriction enzymes and have been successfully used as genome-editing tools, owning to their ability to modify and customize DNA-binding domains by altering transcription in host cells. As DNA targeting proteins, both of them have the ability to regulate the expression of target genes. Moreover, ZFNs and TALENs can recognize any sequence and be tagged to different kinds of effector domains, such as transcriptional activators and repressors, recombinases, transposases, DNA, histone methyl-transferases, and histone acetyltransferases (Fig. 4a, b). Synthetic ZF proteins, which combine serval ZF protein domains, can recognize 9–18 bp specific DNA sequences, while TALE proteins are designed to combine the DNA-binding repeat domains using 7–34 DNA base pairs [74–76]. Compared to TALENs, ZFNs do not require generic targeting sequences, thus allowing great flexibility in the targeting. It also can facilitate both genomic integrations and gene knock-outs [77]. On the contrary, efficient endogenous deletions and gene insertions were recently tended to use TALEN architecture [78]. ZFNs can provide an unprecedented tool for researchers and have been successfully used for HDR mediated gene knock-in (KI) and NHEJ-mediated knock-out (KO) in many gene-editing experiments including eukaryotes as well as prokaryotes [79]. Furthermore, TALENs were more frequently used for the genetic engineering in mammalian cells, insects, vertebrates, diverse plants, and dengue vector [80–82]. However, they also show some disadvantages. For example, they need extensive protein engineering for defining the target specificity of nuclease.

Compared with the above two approaches, CRISPR is a better system, as designing the guide RNA (gRNA) for the target gene is much simpler, less labor intensive, more specific and sensitive [83] (Fig. 4c). In addition, CRISPR/Cas9 has other advantages, such as directional knock-out of genomic DNA, multilevel control of gene expression. In nature, CRISPR systems mainly defend bacteria against foreign invasive DNA by integrating it at the host’s CRISPR locus. The CRISPR–Cas9 complex contains a single CRISPR associated protein (Cas9), two CRISPR RNA (crRNA) and trans-acting antisense CRISPR RNA (trcRNA). It is transcribed and processed into CRISPR RNA (crRNA), which complexes with transactivating CRISPR RNA (tracrRNA) and CRISPR associated protein 9 (Cas9). This complex is guided by crRNA to its target invasive DNA, which is cleaved by the endonuclease activity of the Cas9, thus protect the host. For genome editing, the crRNA and tracrRNA are replaced by gRNA [84]. Cas9 is deactivated (dCas9) to be targeted at the locus of interest in the model organism. However, the need for protospacer adjacent motif (PAM) downstream of the gRNA target sequence in the genome becomes the main design constraint. The ability to direct dCas9 to any desired target by just changing the sequence of the gRNA makes CRISPR–dCas9 a better option over ZFPs and TALEs due to the customized sequence specificity via target specific gRNAs. When fused with the correct effector domains, dCas9 can repress (CRISPRi) or activate (CRISPRa) expression of target genes. A CRISPR-based logic gate was also uploaded in *E. coli* to regulate gene expression, where an inducible promoter drove gRNA expression in response to a small-molecule input. The expressed gRNA recruited dCas9 to the constitutive promoter of an output gene inhibiting RNAP binding to the output promoter. Furthermore, the discovery of CRISPR–Cas9 RNA-guided tools can now be easily utilized for virtual gene modulation of any organism.

Recently, it has been reported that multiple gene integrations into protein network are possible on a larger scale. For example, it was found that integration of multiple copies of genes from the yeast genome at the transcriptional active site may result in stable, uniform, and high production of recombinant proteins [85].

Using endogenous genes of *E. coli* is an approach for single plex genome engineering [12, 86]. RecA, RecF as well as the exonuclease and helicase functions of the RecBCD enzyme complex are commonly used for genome engineering. Moreover, genome engineering using bacteriophage-derived proteins is another available approach, which uses RecET proteins encoded by Rac phage and red proteins encoded by λ phage. It was found that recombination rates of RecBCD and RecF pathways are not high enough for efficient engineering. Therefore, sequential engineering of the genome is performed to replace introduction of DNA cassettes for multiple loci simultaneously. Red proteins from bacteriophage λ can be used for genome engineering, while λ red recombination generally uses three pL promoter-regulated genes [87]. To propel forward genome engineering, multiplex automated genome engineering (MAGE) was presented on the basis of the λ red recombination system [88]. Traditional λ red recombination uses DNA fragments to target a single locus, whereas MAGE uses ssDNA to target multiple loci in *E. coli* genome. MAGE allows more rapid multiplex genome engineering and generates better combinatorial libraries compared to older methods. Particularly, MAGE can simultaneously manipulate numerous target loci and provide combinatorial libraries which can be used as an optimization tool for engineering of complex cellular networks. And also, improvements of generalized screening methods along with MAGE will pave the way to a more efficient approach to genome engineering of valuable bacterial strains [89, 90].

### Application of the advanced techniques in recombination systems

During the last several decades, targeted genome-engineering technology has been developed to investigate various functions of genes in cellular networks. A wide range of organisms have been probed, regulated, and manipulated for the creation of genetic KI, KO, and genetic modifications [91]. For example, *E. coli* and *S. cerevisiae*, the preferred model organisms when researchers investigate genome engineering have offered substantial benefits owing to their available whole genome sequences [92]. The most updated advances using the recombination systems are summarized.

The CRISPR–Cas9 system, originating from prokaryotes, has quickly become a revolutionary tool in genome engineering and made great difference in other organisms including bacteria, yeasts, fungi, plants, and mammalian cells [93–97]. In 2014, Bao et al. optimized CRISPR–Cas9 system for one-step multiple gene disruption in the model organism *S. cerevisiae*. A 100 bp dsDNA mutagenizing homologous recombination donor was inserted between two direct repeats for each target gene in a CRISPR array consisting of multiple donor and guide sequence pairs. This system is composed of a variant of wild-type Cas9, multi-copy plasmids, a homology-integrated crRNA cassette, and tracrRNA. As a result, three genes of *ade2* (encoding phosphoribosylaminoimidazole carboxylase), *can1* (encoding plasma membrane arginine permease), and *lyp1* (encoding lysine permease) were disrupted within 4 days simultaneously with the efficiency ranging from 27 to 87%. Then, another three genes of *gcy1*, *atf2,* and *ypr1* (involved in an artificial hydrocortisone biosynthetic pathway) were disrupted simultaneously within 6 days with 100% efficiency, which demonstrated the high efficiency for multiple gene knock-outs [98]. One year later, an easier and faster strain construction method was developed for simultaneous introduction of multiple genetic modifications in *S. cerevisiae*. This method was consisted of assembled plasmids containing two gRNAs and several genetic modifications. With the aid of web-based identification of suitable Cas9 target sites (http://yeastriction.tnw.tudelft.nl), six genetic modifications were introduced with high efficiency [99]. This system achieved a combination of gene introduction with gene deletion and laid a good foundation for future studies.

To extend the usage of RNA-guided Cas9 as a metabolic pathway building tool, β-carotene biosynthetic pathway of 17 overlapping DNA fragments was successfully introduced into the yeast genome using direct assembly and chromosomal integration method [100]. Tsai et al. reconstructed a xylose utilizing pathway in *S. cerevisiae* using sgRNA/Cas9, and demonstrated the similar performance compared with the strain constructed through traditional integration approaches using selectable markers [101]. A high-efficiency, single-step, markerless and multi-copy method for full biochemical pathway integration was further developed by Shi et al. This research combined xylose utilization pathway and (R, R)-2,3-butanediol (BDO) synthesis pathway, and thus generated a strain that was able to produce BDO directly from xylose [102].

## Optimization of biological system

A series of components were required when building a synthetic biological system, which involves genetic parts, devices, and systems. Designed tools were capable of regulating these components in a predictable and quantitatively controllable manner. Imbalances within systems can lead to the failure of the designed program. Recently, novel genetic tools that can be utilized to design and optimize biological systems have gained more attention. To achieve the optimal manifestation of the designed synthetic biological system, balanced and suitable expressions of multiple enzymes, which constitute a metabolic pathway or genetic program, are essential. Hence, controlling gene expression in a predictable and quantitatively controllable manner is one of the most important design principles.

In general, gene expression can be regulated at three levels: transcriptional, post-transcriptional, and post-translational levels [103]. Transcriptional control of gene expression has been widely used in metabolic engineering to optimize biological systems by creating synthetic promoter libraries with a broad range of transcription efficiencies in diverse prokaryotes and eukaryotes [104]. The increasing number of synthetic promoters and availability of technology for duplication of genes in chromosomes will facilitate the search of a larger solution space and aid in the development of long-term genetically stable strains. During the last two decades, it has been shown that the structural elements around the translation–initiation region (TIR) of mRNA are important in determination of the amount of proteins produced from particular mRNA sequences [105].

Introduction of foreign metabolic pathway into a heterologous host for value-added product production often entails re-optimizing the codons of the target genes based on the usage patterns of the host systems [106]. However, synonymous codon replacement in regions of inherently slow mRNA translation from rare to abundant codons can deleteriously affect three-dimensional structure of protein and enzyme. Because miRNAs are widely implicated in the regulation of gene expression, it is highly likely that their expression/activities influence all cellular processes to a certain extent [107]. Therefore, miRNAs might be well suited as targets for engineering eukaryotic cells by allowing the adjustments of diverse processes, such as metabolism, cell cycle, and apoptosis. Whereas current cell engineering strategies are typically based on modulating expression of effector proteins (e.g., cell cycle regulators, chaperones, functional enzymes, or anti-apoptotic factors) to endow specific cell phenotypes, interfering with miRNA activity completely avoids the potential overcharging of translational machinery.

However, redesigning existing pathways or constructing new metabolic pathways sometimes failed to achieve the original target due to the failure of functionally heterologous genes expression or wrong algorithms. To solve this problem, computer-aided design (CAD) tools for predicting and evaluating genetic manipulations in advance have been developed using an automated genetic compiler, such as genome scale of metabolic network model (GSMM).

## Future and perspectives

Although synthetic biology has not achieved its exact definition, it has brought unprecedented changes to the biotech industry, and was hailed as one of top ten new technologies, which would change the world in the future [108–110]. Especially, technological innovations in the synthesis of nucleic acids and DNA sequencing have decreased the cost of DNA synthesis and accelerated the development of synthetic genomics, making de novo synthesis of genes and even whole genomes possible. New biological functions can be designed and used for research and application purposes. With the development of synthetic genomics, the research object has gradually shifted from the initial virus, bacteria to yeast, fungi, plant, and mammalian cell, whose regulation system is more complex. With the deeper and higher understanding of natural systems, synthetic biology research will shift from the theory to the practical large-scale application, which has the potential to revolutionize biomedicine and biotechnology.

In consideration of increasing environmental concerns and the depletion of fossil fuel reserves, chemicals derived from renewable biomass are considered as the promising alternatives. With the help of cutting-edge genome-editing tools, assembly of sophisticated gene networks, and computer-aided guidance, the highly complex interconnected synthetic genetic circuits and pathways can be designed in bacteria and yeast, providing unprecedented sensor, processing, and production capacity to enable new opportunities in conversion of biomass to value-added biochemicals.

There is high expectation that synthetic biology could make biofuels and biochemicals production more environmentally friendly. However, it has touched off a heated controversy of scientific ethics, legal and social issues while benefiting humans. To facilitate screening, various antibiotic-resistant genes have been introduced in microorganisms, which means that some products of synthetic biology may pose threats to humans and the environment. Therefore, when we encourage and support the development of synthetic biology, the supervision system of synthetic biology should also be strengthened. It would be better to conduct synthetic biology research in collaboration with social scientists, and in a manner of ongoing public engagement to raise awareness on the social issues. Ultimately, if we can do that above, synthetic biology will bring us unimaginable benefits.
